# Modeling and Experimental Testing of an Unmanned Surface Vehicle with Rudderless Double Thrusters

**DOI:** 10.3390/s19092051

**Published:** 2019-05-02

**Authors:** Chunyue Li, Jiajia Jiang, Fajie Duan, Wei Liu, Xianquan Wang, Lingran Bu, Zhongbo Sun, Guoliang Yang

**Affiliations:** 1 The State Key Lab of Precision Measuring Technology and Instruments, Tianjin University, Tianjin 300072, China; 2016202086@tju.edu.cn (C.L.); fjduan@tju.edu.cn (F.D.); wxq1993@tju.edu.cn (X.W.); lingranbu@tju.edu.cn (L.B.); zbsun@tju.edu.cn (Z.S.); guoliang_yang@tju.edu.cn (G.Y.); 2 The Department of Electronic and Electrical Engineering, University of Sheffield, Sheffield S10 2TN, UK; w.liu@sheffield.ac.uk

**Keywords:** unmanned surface vehicles, rudderless double thrusters, propeller thrust model, three-DOF dynamic model, system identification

## Abstract

Motion control of unmanned surface vehicles (USVs) is a crucial issue in sailing performance and navigation costs. The actuators of USVs currently available are mostly a combination of thrusters and rudders. The modeling for USVs with rudderless double thrusters is rarely studied. In this paper, the three degrees of freedom (DOFs) dynamic model and propeller thrust model of this kind of USV were derived and combined. The unknown parameters of the propeller thrust model were reduced from six to two. In the three-DOF model, the propulsion of the USV was completely provided by the resultant force generated by double thrusters and the rotational moment was related to the differential thrust. It combined the propeller thrust model to represent the thrust in more detail. We performed a series of tests for a 1.5 m long, 50 kg USV, in order to obtain the model parameters through system identification. Then, the accuracy of the modeling and identification results was verified by experimental testing. Finally, based on the established model and the proportional derivative+line of sight (PD+LOS) control algorithm, the path-following control of the USV was achieved through simulations and experiments. All these demonstrated the validity and practical value of the established model.

## 1. Introduction

As an intelligent offshore platform, unmanned surface vehicles (USVs) have in the recent years been playing an increasingly significant role in the field of intelligent ships. By carrying a variety of sensors, it is possible for USVs to continuously monitor various marine phenomena such as ocean waves, tides and marine meteorology in replacement of traditional human-controlled ships and operations [1,2]. Given their small size, intelligence, and unmanned nature, they can avoid casualties, and can therefore be applied to antisubmarine and anti-mine warfare, maritime security protection, and other military fields [3,4]. In recent years, although the technological development of environmental sensing, wireless communication, positioning and navigation for USVs has made significant progress, the motion control aspect of USVs has lagged, such as in autonomous navigation and path-following control. It plays an essential role in some marine applications, such as seawater monitoring for fixed points, water sampling for fixed paths and even tracking control of the ordinary merchant ship [5,6,7]. For effective motion control of USVs, it is necessary to establish a mathematical model about the kinematics and kinetics performance of the USVs [8,9], which is the basis for realizing intelligent control and maneuverability prediction.

There are two kinds of mathematical models for ship motion. One is the Abkowitz model [10,11], which is also called the global model. It considers the hull, propeller and rudder as an integrated whole, and expands the hydrodynamic force acting on the hull into the Taylor series of each motion variable. The other is the so-called MMG model [12,13,14], which is also known as the separable model. It was proposed by the Japanese Mathematical Modeling Group (MMG) based on the Abkowitz model. The MMG model decomposes hydrodynamic forces into three parts of the hull, propeller and rudder, and takes the interaction between them into account. The Abkowitz model is mathematically more complete and rigorous, and therefore widely used in the community. Based on the Abkowitz model, Fossen et al. [15,16] put forward a method of modeling and control for marine craft, including ships, high-speed craft, semi-submersibles and floating rigs. They conducted a detailed analysis of the kinematics and kinetics performance of the marine craft. J. Menoyo Larrazabal et al. [17] formulated a coupled surge-sway-yaw model to guide the USV maneuvering control and dealt with the rudder control of a USV system. Based upon a simplified 3 degrees of freedom (DOFs) model and an actuation model, Petr Švec et al. [18] presented a trajectory planning and tracking approach to follow a differentially constrained target vehicle operating in an obstacle field. Simulation and experimental studies were conducted to demonstrate the effectiveness of the developed method.

However, these models are all aimed at USVs with the combination of thrusters and rudders. In the actuator of this kind of USVs, the thrust value of the thruster controls USV velocity, and the rotation angle of rudder controls USV steering. The mechanical structure of a combination of thrusters and rudders is complicated, and the rudder used is often plastic, which easily can be damaged by the wear of the gear during the steering process, and as a result significantly affects the reliability of the USV. To deal with this problem, the structure of the rudderless double thrusters is employed, where the thrust values of port and starboard thrusters control the USV velocity, and the differential thrust controls the steering of the USV. It simplifies the mechanical structure of the USV, and makes the steering process more flexible. The structure reduces the control of the rudder, and improves the reliability of the USV. Besides, the energy conversion rate of the structure is higher than the combined structure of thrusters and rudders. There has been some research focused on USVs with rudderless double thrusters. Justin E. Manley et al. [4] introduced the development of USVs for nearly 15 years. The actuators of AutoCat and ROAZ II USVs both adopted the structure of rudderless double thrusters for hydrological surveys and oceanographic researches [4]. R. Sutton et al. [19,20,21] analyzed the control system of Springer USV with rudderless double thrusters and implemented the nonlinear control of its motion. A. Savvaris et al. [22] presented the development of the C-Enduro USV with rudderless double thrusters and the collision avoidance algorithms for the sensing and avoidance system. However, the modeling of this kind of USV has rarely been studied before. In order to study the motion and dynamic performance and provide guidance for the motion control of the USVs with rudderless double thrusters, the three-DOF dynamic model and propeller thrust model are established for a 1.5 m long, 50 kg USV with rudderless double thrusters. The USV has a double hull configuration and a custom-designed propulsion system, which consists of two propellers. The model parameters are obtained through system identification.

The main contributions of this paper can be summarized as follows:

(1) The three-DOF dynamic model and propeller thrust model are established and combined for a USV with rudderless double thrusters. It guides the modeling of general USVs with rudderless double thrusters.
(2) Since the direction of thrust generated by the propeller is always consistent with the heading of the USV, which makes the USV unstressed in the lateral direction, the three-DOF model simplifies the force analysis and model designing of the USV. It combines the propeller thrust model in which the unknown parameters are reduced from six to two to represent the thrust in more detail.
(3) System identification is utilized to get the parameters of the three-DOF model and propeller thrust model.
(4) Based on the established model, the path-following control of the USV is achieved through PD+LOS control algorithm. It links theory with practice, and provides guidance for the experimental tests.

This paper is organized as follows. The USV used in this paper is a fully automatic sensing (FAS) USV. Its main characteristics are described in Section 2, with emphasis on the propulsion system and the sensing, communication and guidance, navigation, and control (GNC) system. In Section 3, the three-DOF dynamic model and propeller thrust model are derived. In Section 4, the acceleration test, circle test and zigzag test are performed to obtain the model parameters through system identification. The accuracy of the modeling and identification results are verified by experimental testing. The proportional derivative+line of sight (PD+LOS) control algorithm is utilized to realize the path-following of the USV and the results of simulations and experiments are compared in Section 5. PD stands for proportional and derivative control. The PD controller is based on the error of the system, using the proportional and derivative calculations to control the output of the system. Finally, in Section 6, some concluding remarks are given regarding the results shown and possible future work.

## 2. THE FAS-01 USV

**Figure 1:**
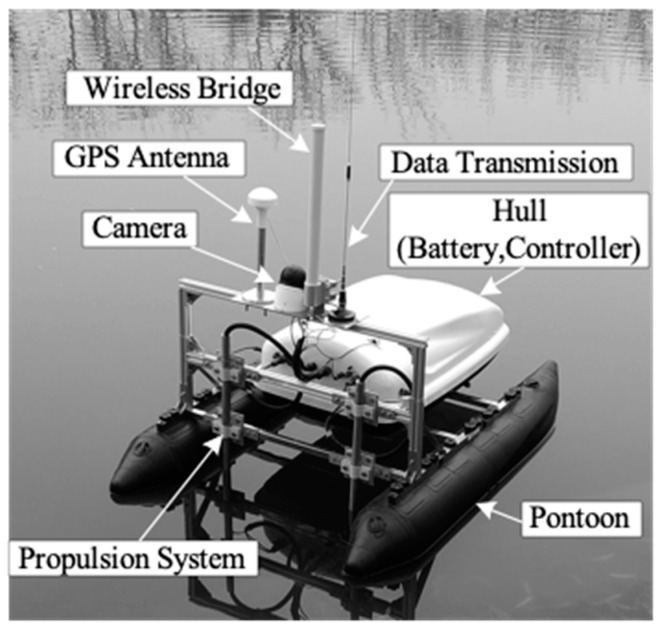
The FAS-01 USV.

The FAS-01 USV designed in this paper is 1.5 m long and weighs 50 kg, as shown in Figure 1. The USV adopts a catamaran structure including two main hulls on the right and left sides symmetrically. The catamaran construction prevents roll and pitch motions and keeps the USV position balanced.

It also affords easy manoeuvrability and stability under excessive load [23,24]. The physical characteristics of the USV are shown in Table 1.

### 2.1. Propulsion System

**Figure 2:**
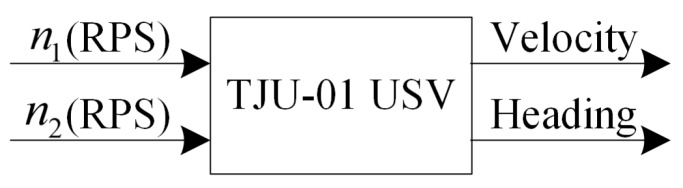
Block diagram of the propulsion system.

The propulsion system guarantees the continuous output of USV power and is an essential part of the USV. It consists of two direct-current (DC) brushless thrusters powered by a 12 V lithium battery. The thrusters are equipped with two three-blade propellers with a diameter of 0.27 m, which are adjusted by two rotary speed controllers, respectively. The USV has a differential steering mechanism and requires two inputs, $n_{1}$ and $n_{2}$ to adjust its heading, where $n_{1}$ and $n_{2}$ are the two propeller speeds in Revolutions Per Second (RPS). The two propeller speeds control the USV velocity, and the differential speed controls the steering of the USV. There is no rudder in the propulsion system, as shown in Figure 2.

Obviously, straight line motion requires port and starboard thrusters to run at the same speed, and the differential thrust is zero in this case [19]. $n_{c}$ and $n_{d}$ represent the common mode and differential mode propeller speeds, respectively. They are defined as:

(1) $$n_{c}=(n_{1}+n_{2})/2,$$

(2) $$n_{d}=(n_{1}-n_{2})/2.$$

The USV with the rudderless double thrusters needs to generate momentum by the differential thrust between the port and starboard propellers to change the heading. During this process, the velocity of the USV also changes. So, there is a coupling between the velocity and the yaw rate. In order to eliminate the effects of coupling and maintain the velocity of the USV, $O_{e}X_{e}Y_{e}$ must remain constant at all times, and $O_{e}X_{e}Y_{e}$ changes around zero depending on the direction of the manoeuvre.

### 2.2. Sensing, Communication and GNC System

Adequate sensing capabilities are generally required to enhance the performance of the USV. The inertial measurement unit (IMU) and global positioning system (GPS) as the basic sensors are typically used in combination with the system to guarantee the USV remaining in good operating condition, and to improve its performance [25]. The IMU/GPS is used to estimate the position and orientation of the USV during sailing. It has a resolution of 1 degree in the heading. The GPS can provide up to 1 m accuracies in both latitude and longitude, depending on cloud cover and satellite availability. Besides, the USV has ultrasonic sensors to provide information on surrounding obstacles. Cameras, radar, sonar, as well as other kinds of sensors are optionally adopted, depending on the specific task at hand.

The communication system includes not only wireless communication with the ground control station and other vehicles to perform cooperative control, but also onboard wired/wireless communication with a variety of sensors, actuators, and other equipment. Therefore, the reliability of the communication system is crucial for USV. Due to the different communication rates and distances required for data and image transmission between the USV and the ground control station, we adopt a different communication scheme. The data transmission uses 433 MHz radio frequency (RF) communication. The image and video transmissions take a 5 GHz wireless bridge. The sensors, actuators, and other equipment are communicated with the microcontroller through universal asynchronous receiver/transmitter (UART) in order to obtain real-time status information of the USV.

As the most vital component of a USV, the GNC system is generally composed of an onboard microcontroller and some algorithms. Firstly, the GNC system obtains the past and current states of the USV (such as position, orientation, velocity, and acceleration), and the environmental information including the ocean currents and wind speed from the onboard sensors. Secondly, based on actual environmental conditions and task requirements, it generates a series of desired paths and points or obtains the desired paths from the ground station. Finally, the GNC system produces the control command to the actuator based on control algorithms to complete the specified steering and forward and achieve path-following and autonomous navigation of the USV.

## 3. Three-DOF Dynamic Model and Propeller Thrust Model

### 3.1. Three-DOF Dynamic Model

The availability of a sufficiently accurate USV model enabling effective control design is imperative for both control methodology design and simulation study purposes [24]. This requires a prior investigation of both a precise mathematical USV model with reasonable system parameters. Generally, the study of a standard USV dynamic model can be divided into two parts: kinematics, which treats only geometrical aspects of motion, and kinetics, which is the analysis of the forces causing the motion.

#### 3.1.1. Kinematic

**Figure 3:**
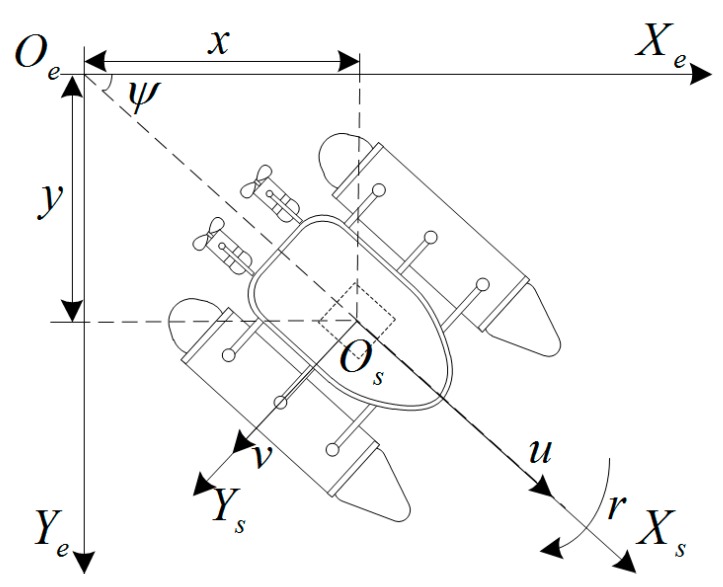
Schematic diagram of three DOF of the USV.

Within the field of kinematics, an ocean vessel moves in six DOFs, which are defined by: surge, sway, heave, roll, pitch, and yaw. However, the six-DOF model is complicated. Because some DOFs in the USV model have inherent stability, the model can be simplified. The buoyancy of the USV stabilizes the regular motion so that heave can be ignored. Because of the sufficient longitudinal and lateral metacentric height of the USV, the motion of roll and pitch can also be ignored. Therefore, the six-DOF model can be simplified to a three-DOF model to describe the planar motions of USV in surge, sway and yaw [25], as shown in Figure 3.

To determine the equations of motion, two reference coordinate systems are considered: the inertial or fixed to earth frame $O_{e}X_{e}Y_{e}$ that may be taken to coincide with the USV fixed coordinates in some initial condition and the body-fixed frame $O_{s}X_{s}Y_{s}$ in Figure 3. Since the motion of the earth hardly affects the USV (different from air vehicles), the earth-fixed frame $O_{e}X_{e}Y_{e}$ can be considered to be inertial. The body axes $O_{s}X_{s}$ and $O_{s}Y_{s}$ coincide with the principal axes of inertia and are usually defined as follows: $O_{s}X_{s}$ is the longitudinal axis (directed from aft to fore); $O_{s}Y_{s}$ is the transverse axis (directed to starboard).

The typical USV kinematic model [16] in planar motion and without the presence of disturbances can be expressed as:

(3) $$\dot{\eta }=J(\eta )\upsilon$$

where $\eta ={\left[\begin{array}{ccc} x & y & \psi \end{array}\right]}^{T}$ is the position (x,y) and yaw (ψ) of the USV in the earth-fixed frame, $\dot{\eta }={\left[\begin{array}{ccc} \dot{x} & \dot{y} & \dot{\psi } \end{array}\right]}^{T}$ describes the vehicle linear North($\dot{x}$), East($\dot{y}$), and Z-axis angular velocities($\dot{\psi }$) in the earth-fixed frame, $\upsilon ={\left[\begin{array}{ccc} u & v & r \end{array}\right]}^{T}$ is the vehicle surge velocity (*u*), sway velocity (*v*) and yaw rate (*r*) and the transformation matrix J(η), related to yaw (ψ), is given by:

(4) $$J(\eta )=\left[\begin{array}{ccc} \cos\psi & -\sin\psi & 0\\ \sin\psi & \cos\psi & 0\\ 0 & 0 & 1 \end{array}\right]$$

#### 3.1.2. Kinetic

In addition to the kinematic model, it is also necessary to analyze the force of the USV to set up the kinetic model. When designing the control system of the USV, there are clear advantages using the vectorial model [5] instead of the component forms of the Taylor-series expansions in the Abkowitz model [8,9]. The main reasons are that system properties such as symmetry, skew-symmetry and positiveness of matrices can be incorporated into the stability analysis [16]. The kinetic model of the USV is given by

(5) $$M\dot{\upsilon }+C(\upsilon )\upsilon +D(\upsilon )\upsilon =\tau +\tau_{E}$$

(6) $$M=\left[\begin{array}{ccc} m-X_{\dot{u}} & 0 & -my_{g}\\ 0 & m-Y_{\dot{v}} & mx_{g}-Y_{\dot{r}}\\ -my_{g} & mx_{g}-N_{\dot{v}} & I_{z}-N_{\dot{r}} \end{array}\right]$$

(7) $$C(\upsilon )=\left[\begin{array}{ccc} 0 & 0 & -m(x_{g}r+v)+Y_{\dot{v}}v+\frac{Y_{\dot{r}}+N_{\dot{v}}}{2}r\\ 0 & 0 & (m-X_{\dot{u}})u\\ m(x_{g}r+v)-Y_{\dot{v}}v-\frac{Y_{\dot{r}}+N_{\dot{v}}}{2}r & -(m-X_{\dot{u}})u & 0 \end{array}\right]$$

(8) $$D(\upsilon )=D+D_{n}\left(\upsilon \right)\;=-\left[\begin{array}{ccc} X_{u} & 0 & 0\\ 0 & Y_{v} & Y_{r}\\ 0 & N_{v} & N_{r} \end{array}\right]-\left[\begin{array}{ccc} X_{u\left|u\right|}\left|u\right| & 0 & 0\\ 0 & Y_{v\left|v\right|}\left|v\right|+Y_{v\left|r\right|}\left|r\right| & Y_{r\left|v\right|}\left|v\right|+Y_{r\left|r\right|}\left|r\right|\\ 0 & N_{v\left|v\right|}\left|v\right|+N_{v\left|r\right|}\left|r\right| & N_{r\left|v\right|}\left|v\right|+N_{r\left|r\right|}\left|r\right| \end{array}\right]$$

where M is the mass matrix, and $x_{g}$ and $y_{g}$ are the coordinates of the USV center of gravity in the body-fixed frame. The model designates the body-fixed origin at the center of gravity and assumes horizontal symmetry, leading to $x_{g}=0$ and $y_{g}=0$; $I_{z}$ denotes the moment of inertia about the Z_s_ -axis. The notation of SNAME [26] for hydrodynamic derivatives is used in this expression. For instance, the hydrodynamic added mass force Y along the *y*-axis due to the acceleration $\dot{u}$ in the *x*-direction is written as

(9) $$Y=-Y_{\dot{u}}\dot{u}\;\;\;\;Y_{\dot{u}}:=\partial Y/\partial \dot{u}$$

where C(υ) is the Coriolis and centripetal matrix, D(υ) is the drag matrix, including the linear drag term (D) and the nonlinear drag term ($D_{n}\left(\upsilon \right)$), **τ** is the vector of forces and moments generated by the propulsion system, and $\tau_{E}={\left[\begin{array}{ccc} \tau_{uE} & \tau_{vE} & \tau_{rE} \end{array}\right]}^{T}$ is the vector of forces and moment caused by the disturbance.

For the USV with rudderless double thrusters, the thrust generated by the port and starboard thrusters is always in the same direction as the heading of the USV, and there is no force generated by the rudder. Therefore, the vector **τ** can be expressed as

(10) $$\tau ={\left[\begin{array}{ccc} \tau_{u} & 0 & \tau_{r} \end{array}\right]}^{T}$$

where $\tau_{u}=X_{P1}+X_{P2}$. $X_{P1},\;X_{p2}$ denote the thrust from the port and starboard thrusters, respectively.

Torque by the port and starboard thrusters is given by

(11) $$M_{r}=X_{P1}\times (l_{p}i+d_{p}j)+X_{P2}\times (-l_{p}i-d_{p}j)$$

where i and j are unit vectors in the $X_{e}$ and $Y_{e}$ directions. $d_{P}$ is the transverse distance from the centerline of the USV to the centerline of each thruster, which equals 0.26 m in the paper. $l_{p}$ is the longitudinal distance from the thruster to the center of gravity. Because the direction of the torque by the port and starboard thrusters is both in the Z_s_ -axis, to simplify the calculation, the scalars of the torque are taken in the model. $\tau_{r}$ is the magnitude of $M_{r}$, that can be expressed as;

(12) $$\tau_{r}=(X_{P1}-X_{P2})\cdot \sqrt{l_{p}{}^{2}+d_{p}{}^{2}}\frac{d_{p}}{\sqrt{l_{p}{}^{2}+d_{p}{}^{2}}}=(X_{P1}-X_{P2})\cdot d_{P}$$

It can be seen that the propulsion of the USV is provided entirely by the resultant force generated by the port and starboard thrusters and that the rotational moment is related to the differential thrust and the distance of two thrusters. This cancels the function of the rudder to make the calculation simpler. Modeling analysis of the propeller thrust is detailed in the next part.

In order to simplify controller design, we make the following assumptions of the kinetic model:

(1) When the USV is slow (the maximum velocity of the USV is 1.5 m/s.), the effect of nonlinear drag term ($D_{n}\left(\upsilon \right)$) can be ignored.
(2) Because the effect of off-diagonal terms on the USV’s dynamics is smaller than the diagonal terms, the off-diagonal terms of M and D can be ignored.
(3) Assuming the coincident center of added mass and gravity $N_{\dot{v}}$ can be replaced by $Y_{\dot{r}}$. A combination of approximate fore-aft symmetry and light draft suggests that the sway force arising from yaw rotation and the yaw moment induced by the acceleration in the sway direction are much smaller than the inertial and added mass terms. Therefore, in the Coriolis and centripetal matrix C(υ), $N_{\dot{v}}=Y_{\dot{r}}=0$.
(4) Assuming the USV sails in a calm environment, the environmental disturbances $\tau_{E}$ can be neglected.

Based on these assumptions, the kinetic model can be simplified to

(13) $$M\dot{\upsilon }+C(\upsilon )\upsilon +D(\upsilon )\upsilon =\tau$$

(14) $$M=\left[\begin{array}{ccc} m-X_{\dot{u}} & 0 & 0\\ 0 & m-Y_{\dot{v}} & 0\\ 0 & 0 & I_{z}-N_{\dot{r}} \end{array}\right]$$

(15) $$C(\upsilon )=\left[\begin{array}{ccc} 0 & 0 & -(m-Y_{\dot{v}})v\\ 0 & 0 & (m-X_{\dot{u}})u\\ (m-Y_{\dot{v}})v & -(m-X_{\dot{u}})u & 0 \end{array}\right]$$

(16) $$D(\upsilon )=D=-\left[\begin{array}{ccc} X_{u} & 0 & 0\\ 0 & Y_{v} & 0\\ 0 & 0 & N_{r} \end{array}\right]$$

Therefore, the three-DOF model of the USV with rudderless double thrusters can be expressed by

(17) $$\{\begin{array}{c} \dot{\eta }=J(\eta )\upsilon \\ M\dot{\upsilon }+C(\upsilon )\upsilon +D(\upsilon )\upsilon =\tau \end{array}$$

where $\tau ={\left[\begin{array}{ccc} X_{P1}+X_{P2} & 0 & (X_{P1}-X_{P2})\cdot d_{P} \end{array}\right]}^{T}$. The expanded form of three-DOF equations of motion is:

(18) $$\{\begin{array}{c} \dot{x}=u\cos\psi -v\sin\psi \\ \begin{array}{c} \dot{y}=u\sin\psi +v\cos\psi \\ \begin{array}{c} \dot{\psi }=r\\ (m-X_{\dot{u}})\dot{u}-(m-Y_{\dot{v}})vr+X_{u}u=X_{P1}+X_{P2} \end{array}\\ (m-Y_{\dot{v}})\dot{v}+(m-X_{\dot{u}})ur+Y_{v}v=0 \end{array}\\ (I_{z}-N_{\dot{r}})\dot{r}-((m-X_{\dot{u}})-(m-Y_{\dot{v}}))uv+N_{r}r=(X_{P1}-X_{P2})\cdot d_{p} \end{array}$$

By designing the three-DOF model of the USV with rudderless double thrusters, it reduces the analysis of the force generated by the rudder. Since the direction of thrust generated by the propeller is always consistent with the heading of the USV, which makes the USV unstressed in the lateral direction, it also simplifies the force analysis and model designing of the USV.

### 3.2. Propeller Thrust Model

The thrust generated by the rudderless double thrusters can be decomposed into two forces: the longitudinal force to keep the USV moving forward and the steering moment to change the heading of the USV. In USVs with a combination of thrusters and rudders, the steering moment is the effect of the rudders. However, in this paper, the USV is not equipped with a rudder. The differential thrust generated by double thrusters produces the steering moment. It simplifies the mechanical structure of the USV, and makes the steering of the USV more flexible. Besides, it greatly improves the reliability and energy conversion rate of the USV.

The propeller thrust model can be expressed as [25]

(19) $$X_{P}=(1-t_{P})\rho D^{4}K_{T}(J_{0})n\left|n\right|$$

where ρ is water density, D is the diameter of the propeller, and $t_{P}$ represents the coefficient of thrust reduction. When the propellers are working behind the USV, the water flow velocity at the stern is increased due to the suction effect, resulting in an increase in the hull pressure resistance and frictional resistance. The thrust portion used to offset this increased resistance ΔT is the thrust deduction $t_{P}=(X_{P}-\Delta T)/X_{P}$. The advance ratio $J_{0}$ is given by

(20) $$J_{0}=V(1-\omega )/(nD)$$

where $V=\sqrt{u^{2}+v^{2}}$ is the velocity of the USV, ω is the wake fraction number with ω<1 accounting for the relative reduction in flow velocity into the propeller due to the USV’s wake, and *n* is the propeller speed. In Equation (17), $K_{T}$ is a thrust coefficient depending on the advance ratio $J_{0}$ which is well-modeled as a linear function [27,28], given by

(21) $$K_{T}=\alpha_{1}+\alpha_{2}J_{0}$$

where $\alpha_{1}$ and $\alpha_{2}$ are two constants.

Based on Equations (19–21), the propeller thrust $X_{P}$ can be expressed as

(22) $$X_{P}=cVn+d\left|n\right|n$$

where $c=(1-t_{P})(1-\omega )\rho D^{3}\alpha_{2}$, and $d=(1-t_{P})\rho D^{3}\alpha_{2}$.

From Equation (22), we can obtain the propeller thrust model by identifying two parameters *c* and *d*. It is simpler than identifying six parameters $\omega ,\rho ,D,\alpha_{1},\alpha_{2},t_{P}$ in Equations (19–22). This simplifies the model and reduces the workload of system identification.

## 4. System Identification and Experimental Validation

The goal of system identification is to obtain a simplified understanding of how the vehicle behaves, so that the control algorithm can accurately calculate control outputs to produce the desired vehicle response. System identification that requires a specific set of input time histories, extracts thrust and hydrodynamic parameters from standard motion measurements, which include the acceleration test, circle test and zigzag test [29,30]. These tests are all open loop maneuverability tests. The data can be obtained from these tests by setting the speeds of the port and starboard propellers manually. System identification results are presented in this section and the three-DOF dynamic model and the propeller thrust model are determined.

### 4.1. Thruster Performance Experiment

In order to achieve motion control of the USV, we need to know the relationship between motor commands and the thrust on the USV. For this test, the USV was tied to a tension meter, connected to a fixed pole. Port and starboard thrusters were controlled by the same motor commands to apply the thrust uniformly in the surge direction. When the line connecting the tension meter and the USV was tight, that is, when the tension was stable, the tension meter reading could be obtained. The reading was the total thrust of the propulsion system. Three experiments were performed under the same experimental conditions, and the average of three readings of the tension meter was used as the final experimental result.

**Figure 4:**
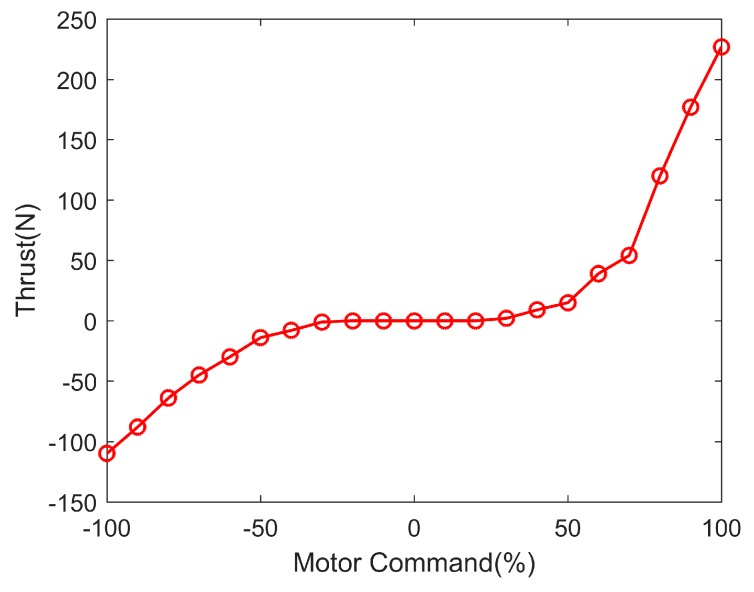
The relationship between motor commands and thrust.

The corresponding thrust under different motor commands is listed in Table 2, and the relationship between motor commands and thrust is shown in Figure 4.

From Figure 4, when the motor commands are from −30% to 30%, the thrust of port and starboard thrusters is zero, which can be called the “dead zone”. As shown in Table 2, we can find that under the same motor commands, the propeller thrust is different when the propeller rotates forward and reverse, and the thrust is larger under the forward rotation than the reverse rotation. The different performance of the thrusters makes the velocity and steering ability different when moving forward and backward.

### 4.2. Propeller Thrust Model Identification

In straight line motion v=r=0, V=u, and the three-DOF dynamic model can be simplified to a one-DOF model [27]. According to the propeller thrust model in Section 3, the one-DOF model is given by:

(23) $$(m-X_{\dot{u}})\dot{u}-(m-Y_{\dot{v}})vr+X_{u}u=X_{P1}+X_{P2}$$

When applying the same motor commands to the port and starboard thrusters, the thrusts of them are the same. Because the performance of port and starboard thrusters is the same, in straight line motion, the thrust $X_{P1}=X_{P2}$, $X_{P1}+X_{P2}=2X_{P}$. Then, Equation (21) can be further expressed as:

(24) $$\dot{u}=\frac{X_{u}}{\left(m-X_{\dot{u}}\right)}u+\frac{2X_{P}}{\left(m-X_{\dot{u}}\right)}$$

When the USV is in the steady state with constant velocity $u_{0}$,

(25) $$0=\frac{X_{u}}{\left(m-X_{\dot{u}}\right)}u_{0}+\frac{2X_{P0}}{\left(m-X_{\dot{u}}\right)}$$

substituting perturbation values $u=u_{0}+\Delta u$, $X_{P1}=X_{P0}+\Delta X_{P}$ into Equation (22),

(26) $$\Delta \dot{u}=\frac{X_{u}}{\left(m-X_{\dot{u}}\right)}(u_{0}+\Delta u)+\frac{2(X_{P0}+\Delta X_{P})}{\left(m-X_{\dot{u}}\right)}$$

According to Equation (23), in the initial limit,

(27) $$\dot{u}(0)=2\frac{\Delta X_{p}}{\left(m-X_{\dot{u}}\right)}=2\frac{c\Delta u\Delta n+d\left|\Delta n\right|\Delta n}{\left(m-X_{\dot{u}}\right)}$$

When the USV is stationary, i.e., *u*_0_ = 0, (25) can be simplified to

(28) $$\dot{u}(0)=\tilde{c}un+\tilde{d}\left|n\right|n$$

where: $\tilde{c}=\frac{2c}{\left(m-X_{\dot{u}}\right)},\tilde{d}=\frac{2d}{\left(m-X_{\dot{u}}\right)}$.

**Figure 5:**
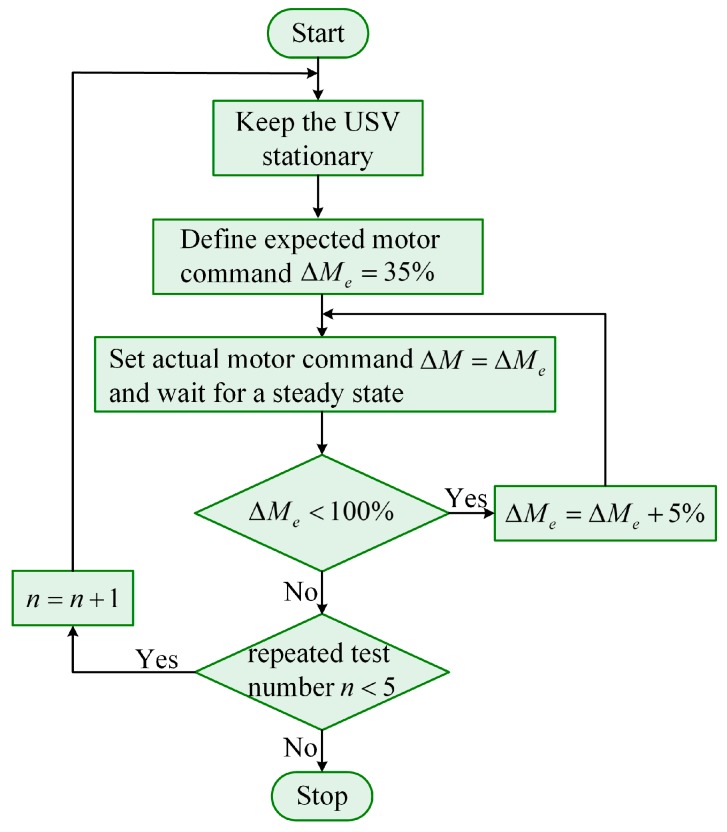
The flow diagram of the test for collecting initial acceleration, surge velocity and propeller speed.

Therefore, through collecting initial acceleration, surge velocity and propeller speed, we can obtain the parameters $\tilde{c}$ and $\tilde{d}$ based on the recursive least squares method. The detailed steps are shown in Figure 5. The initial value of repeated test number n is 0.

**Figure 6:**
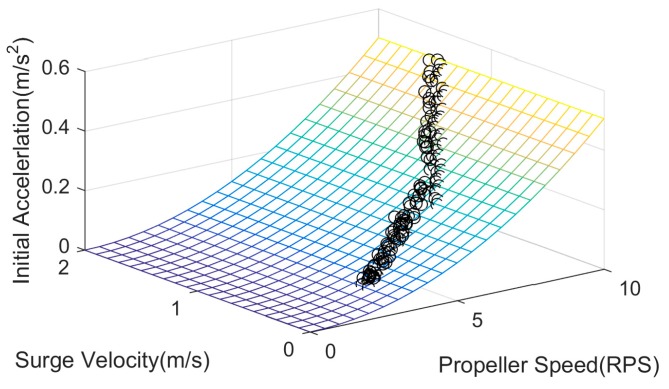
Relationship of initial acceleration, surge velocity, and propeller speed.

Initial acceleration, surge velocity and propeller speed were recorded during execution of the steps to extract $\tilde{c}$ and $\tilde{d}$ in Equation (28). Figure 6 shows the fit of the relationship among the initial acceleration, surge velocity, and propeller speed, where it can be seen that the fitting result is satisfactory when $\tilde{c}=-6.402\times 10^{-6}(s^{-1}\cdot {\mathrm{RPS}}^{-1}\cdot {\mathrm{kg}}^{-1})$, $\tilde{d}=2.014\times 10^{-4}({\mathrm{ms}}^{-2}\cdot {\mathrm{RPS}}^{-2}\cdot {\mathrm{kg}}^{-1})$. Thus, we can obtain the propeller thrust parameters *c* and *d* further.

### 4.3. Three-DOF Model Identification

Using the estimate of the added mass terms presented in [31,32], the hydrodynamic added mass terms in the three-DOF model can be expressed as:

(29) $$m_{11}=m-X_{\dot{u}}\approx m+0.05m=50.05\mathrm{kg}$$

(30) $$m_{22}=m-Y_{\dot{v}}\approx m+0.5(\rho \pi D^{2}L)=84.36\mathrm{kg}$$

(31) $$m_{33}=I-N_{\dot{r}}\approx \frac{m(L^{2}+W^{2})+0.5(0.1mB^{2}+\rho \pi D^{2}L^{3})}{12}=17.21\mathrm{kg}$$

According to the empirical value of the added mass, the propeller thrust parameters can be obtained as follows:

(32) $$c=\tilde{c}(m-X_{\dot{u}})/2=-1.60\times 10^{-4}(s^{-1}\cdot {\mathrm{RPS}}^{-1})$$

(33) $$d=\tilde{d}(m-X_{\dot{u}})/2=5.04\times 10^{-3}({\mathrm{ms}}^{-2}\cdot {\mathrm{RPS}}^{-2})$$

In order to identify the unknown hydrodynamic parameters $X_{u},Y_{v},N_{r}$ in the three-DOF model, a series of experimental tests were conducted for system identification, including the acceleration test, circle test and zigzag test.

**Figure 7:**
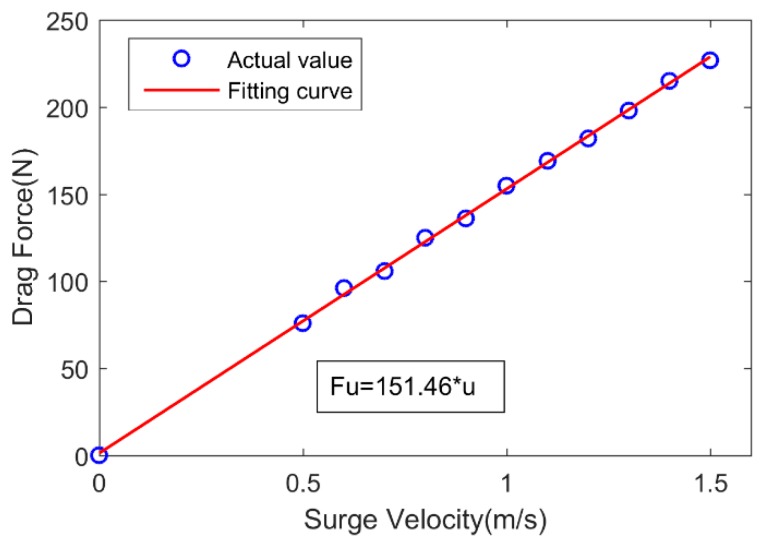
Relationship of surge velocity and drag force in surge direction for USV.

In order to estimate the linear drag coefficient $X_{u}$ in the surge direction, the acceleration test was conducted. The USV was accelerated from the velocity of zero to a different velocity under the specified motor command, and sailed at a steady-state velocity for about 60 s. The range of motor command was 60% to 100%. When the USV achieved steady-state velocity, the drag force $F_{u}=X_{u}u$ in the surge direction was equal to the thrust. Therefore, we could obtain $X_{u}=151.46$ by fitting the relationship of the surge velocity and the drag force, as shown in Figure 7.

**Figure 8:**
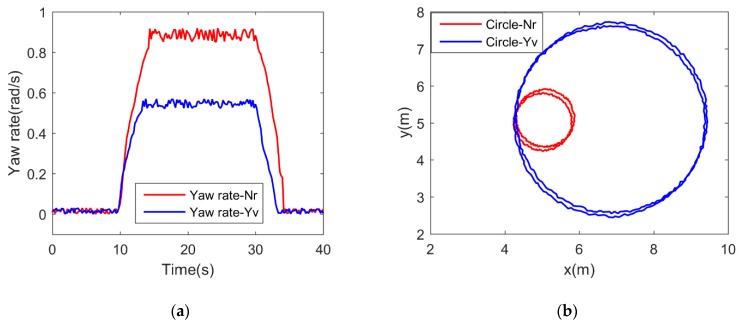
The experimental results of the circle test: (**a**) the yaw rates during circle tests; (**b**) the circle paths for estimating $N_{r}$ and $Y_{v}$ in circle tests.

The circle test was conducted by first establishing a steady state condition and then setting port and starboard motors to different speeds. The USV began tracking a circular pattern in the absence of wind and current for open loop testing for a minimum of 360 degrees. However, it was recommended to conduct the test for 720 degrees to assess environmental effects. This test was conducted at several motor speeds for both port and starboard turns. First, the port and starboard motor commands were set to 100% for 10 s to keep the USV stationary in maximum surge velocity. Then, both motor commands were set to −100% and 100% for 20 s, respectively. In this test, the USV was able to spin in a circle around its center of gravity with minimal surge and sway velocities, which were approximately zero. Therefore, the kinetic model of USV in yaw direction can be simplified to $N_{r}r=(X_{P1}-X_{P2})\cdot d_{p}$. The yaw rate (*r*) was recorded in Figure 8a. The drag moment coefficient from yaw rate $N_{r}$ can then be estimated. The rotation radius is 0.77 m, which is about half of the length of the USV. Similarly, when the USV was stationary in maximum surge velocity, the port and starboard motor commands were set to 0% and 100% for 20 s, respectively. In this test, the USV was able to steer around a turning radius. The acceleration $\dot{v}$ in sway direction, the surge velocity *u*, the sway velocity *v* and the yaw rate *r* were recorded. The yaw rate (*r*) was recorded in Figure 8a. According to the kinetic model in sway direction $(m-Y_{\dot{v}})\dot{v}+(m-X_{\dot{u}})ur+Y_{v}v=0$, the drag coefficient in the sway direction from sway velocity $Y_{v}$ was then estimated. The rotation radius is about 2.56 m. The circle paths for estimating $N_{r}$ and $Y_{v}$ were shown in Figure 8b. Due to the environmental disturbances, such as wind waves and water currents, there are deviations in the circle paths in the first and the second 360 degrees. As shown in Figure 8b, the maximum deviations of the circle path for estimating $N_{r}$ and $Y_{v}$ are 0.055 m and 0.071 m, respectively. The deviations can be ignored relative to the rotation radiuses of the two circle paths.

Combining the identification result of the propeller thrust model, the propeller thrust $X_{P1}$ and $X_{P2}$ in the three-DOF model can be expressed as:

(34) $$X_{pi}=-1.60\times 10^{-4}\sqrt{u^{2}+v^{2}}n_{i}+5.04\times 10^{-3}\left|n_{i}\right|n_{i},\;\;i=1,2$$

Then the three-DOF model identification is achieved, given by:

(35) $$\{\begin{array}{c} \dot{x}=u\cos\psi -v\sin\psi \\ \begin{array}{c} \dot{y}=u\sin\psi +v\cos\psi \\ \begin{array}{c} \dot{\psi }=r\\ 50.05\dot{u}-84.36vr+151.57u=-1.60\times 10^{-4}\sqrt{u^{2}+v^{2}}(n_{1}+n_{2})+5.04\times 10^{-3}(\left|n_{1}\right|n_{1}+\left|n_{2}\right|n_{2}) \end{array}\\ 84.36\dot{v}+50.05ur+132.5v=0 \end{array}\\ 17.21\dot{r}-(50.05-84.36)uv+34.56r=(-1.60\times 10^{-4}\sqrt{u^{2}+v^{2}}(n_{1}-n_{2})+5.04\times 10^{-3}(\left|n_{1}\right|n_{1}-\left|n_{2}\right|n_{2}))\cdot 0.26 \end{array}$$

A zigzag test was conducted to evaluate the model by comparing experimental data with simulations. For the USV with rudderless double thrusters, the zigzag test cannot be carried out through setting up the rudder angle, like 20°/−20°, 10°/−10°. The test should be conducted by varying propeller speeds [30]. Moreover, this is similar to a 20°/−20° zigzag test, except that the angles may be different. The test adopted five combinations of motor commands, as shown in Table 3.

**Figure 9:**
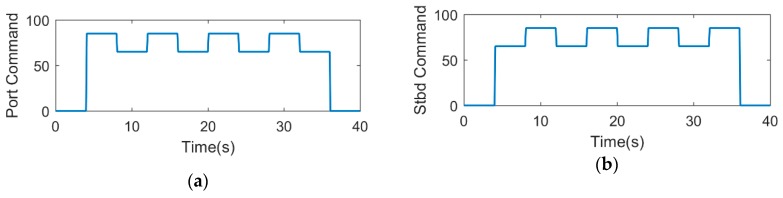
An example of port and starboard motor commands (85%–65%): (**a**) port motor command (85%–65%); (**b**) starboard motor command (65%–85%).

**Figure 10:**
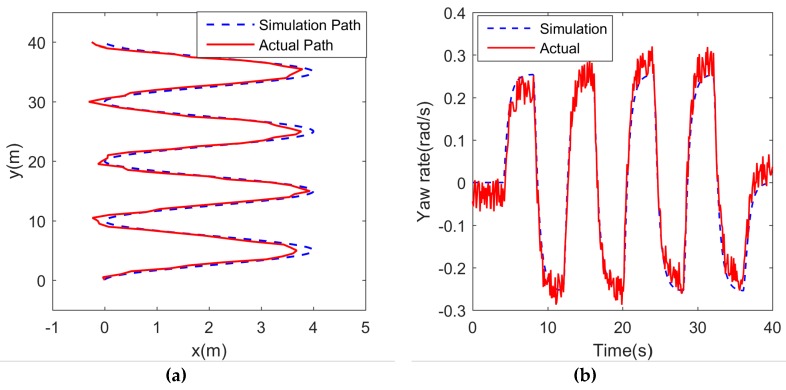
The experimental results of the zigzag test: (**a**) the zigzag simulation path and actual path; (**b**) the result of simulation and actual yaw rate in zigzag test.

Each alternating differential thrust motor command combination was executed with a period of 8 s in a series of 32 s runs. An example of the port and starboard motor commands is shown in Figure 9. Figure 10 is the experimental results of the zigzag test, while Figure 10a is the comparison result between the simulation path and the actual path. It can be seen from Figure 10a that the maximum distance error between the actual path and simulation path is about 0.4 m, which may be due to environmental disturbances. The yaw rate is an important parameter when conducting the zigzag test because it is later used in model development, specifically in determining the maneuvering coefficients pertaining to how fast the USV changes heading. Figure 10b shows the result for both the simulation and actual yaw rates, where they match each other well, verifying the correctness of the model and the identification results.

**Figure 11:**
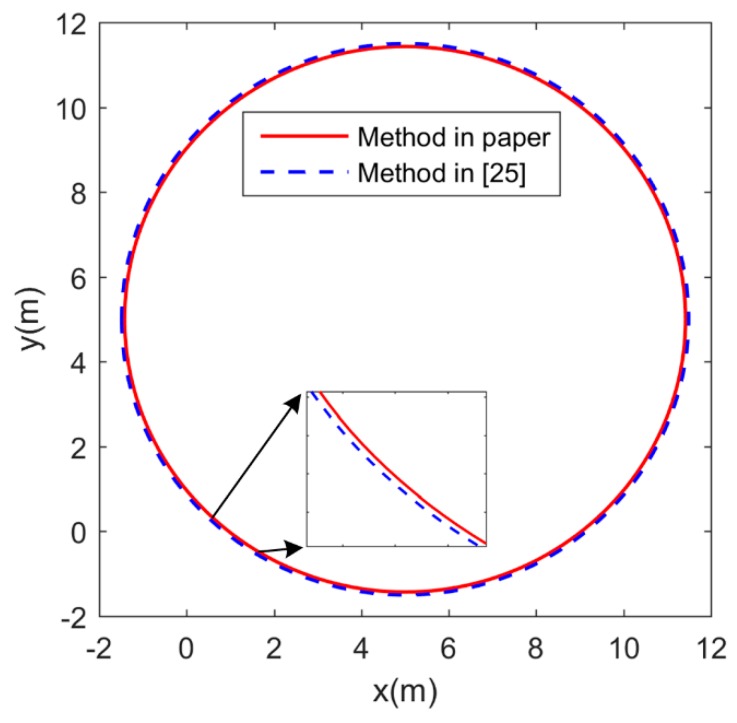
The simulation results of a circle test of the two identification methods.

Based on zigzag test, the parameters in the three-DOF model were identified as an USV with vector propulsion [25]. It mainly used the data u, v, r, n and δ in the zigzag test. δ was the rudder angle. Based on the sampling time, $\dot{u}$, $\dot{v}$ and $\dot{r}$ were calculated. The recursive least squares method was used to identify the parameters of the model. However, in the system identification method in the paper, the acceleration test and circle test were conducted to obtain data for identifying the model parameters. The established model was simplified to one DOF in each test, which made the amount of data small and the calculation simple. A circle test was conducted to compare the two models identified by the identification method in the paper and the method in [25]. The port and starboard motor commands were set to 85% and 55% for 20 s, respectively. The simulation results of a round of the two identification method are shown in Figure 11. It can be seen from Figure 11 that the result of the circle test based on the identification method in the paper is similar to the result based on the method in [25]. It demonstrates that the accuracy of the identification method in the paper is the same as the method in [25]. Therefore, the identification method in the paper can not only ensure the accuracy, but also has lower computational load computation complexity than the method in [25].

## 5. PD+LOS Control Algorithm

For the USV motion control system, the design of the path-following controller is an important part. Due to the simple structure and functional stability of PD control, it is currently used more in autonomous navigation of the ship. Based on the established USV model, the USV motion control system through PD+LOS guidance is designed [33,34,35].

**Figure 12:**
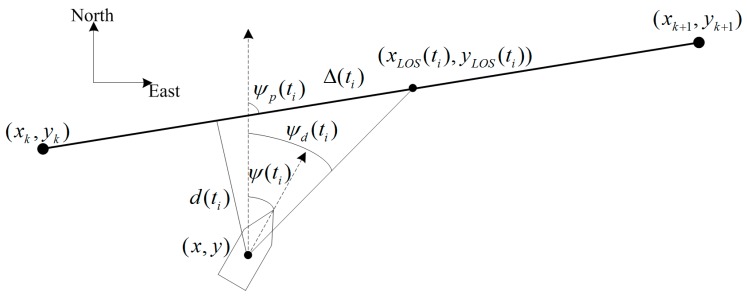
The principle of the LOS guidance algorithm.

The advantages of the LOS algorithm lie in that it is light in computation and easy for implementation. The main principle of the LOS guidance algorithm is to imitate the behavior of a helmsman, which steers the vehicle towards a lookahead distance ahead of the projection point of the vehicle along the path, as shown in Figure 12. The USV assumes a tracking target on the tracking path and then sails along the line of the USV and the hypothetical tracking target. As the USV approaches the desired path, the heading deviation is slowly reduced, and the desired path can be accurately tracked. In Figure 12, $\left(x_{k},y_{k}\right)$ and $\left(x_{k+1},y_{k+1}\right)$ are two adjacent desired waypoints, and the line connecting them is the desired path. (x,y) is the actual position of USV measured by GPS. $\psi (t_{i})$ is the actual heading measured by inertial measurement units (IMU) or compass. $\left(x_{LOS}(t_{i}),y_{LOS}(t_{i})\right)$ is the hypothetical tracking target. The line connecting $\left(x_{LOS}(t_{i}),y_{LOS}(t_{i})\right)$ and the USV is the “line of sight”. The angle between the “line of sight” and the north direction is the desired heading of the USV, $\psi_{d}(t_{i})$, given by

(36) $$\psi_{d}(t_{i})=\psi_{p}(t_{i})+\psi_{r}(t_{i})$$

where $\psi_{p}(t_{i})$ is the path-tangential angle. $\psi_{r}(t_{i})$ is the velocity-path relative angle, $\psi_{r}(t_{i})=\arctan(-d(t_{i})/\Delta (t_{i}))$ It ensures that the velocity is directed toward a point on the path that is located a lookahead distance $\Delta (t_{i})>0$ ahead of the direct projection of $\left(x_{k},y_{k}\right)$ on to the path [36,37,38]. The lookahead distance $\Delta (t_{i})$ is dependent on the cross-track error. This results in lower values for $\Delta (t_{i})$ (and thus a more agile and aggressive response) when the USV is far from the desired path, and greater $\Delta (t_{i})$ values when the USV is closer to the path and less abrupt behavior is needed so as to avoid oscillating around the path. The selection of $\Delta (t_{i})$ refers to [36].

According to the Equation (17), the control law for the USV with rudderless double thrusters can be expressed as

(37) $$M(J\dot{(}\eta {)}^{-1}\dot{\eta }+J(\eta )\ddot{\eta })+C(\upsilon )(J(\eta {)}^{-1}\dot{\eta })+D(\upsilon )(J(\eta {)}^{-1}\dot{\eta })=\tau$$

where $\eta ={\left[\begin{array}{ccc} x & y & \psi \end{array}\right]}^{T}$ is related to the position (x,y) and yaw (ψ) of the USV. The input of the controller is the heading error between the yaw (ψ) and desired heading $\psi_{d}$, which is influenced by η. The controller adjusts the actual heading ψ and the position (x,y) of the USV by controlling the heading error. The Coriolis and centripetal matrix C(υ), the drag matrix D(υ) and the vector of forces and moments generated by the propulsion system τ will be impacted further, which can be seen from Equations (15)–(18). It also realizes the control of the port and starboard propeller thrust through the control of the velocity of the USV and the speeds of two thrusters according to Equation (22) in the established propeller thrust model. Through changing the force and moment of the USV, the controller can achieve the motion control of the USV.

For adopting the PD controller, on the one hand, at first, a proportional integral derivative (PID) controller was carried out in the experimental testing. However, because of $\tau ={\left[\begin{array}{ccc} \tau_{u} & 0 & \tau_{r} \end{array}\right]}^{T}$ in the USV with rudderless double thrusters, the USV was not stressed in the sway direction. It was found that the lateral positioning errors were relatively large due to the less control effect in the sway direction. Under the influence of integral effect, the lateral positioning errors were accumulated, which resulted in that the integral effect was strong and the desired control forces and moments skewed towards regulating the lateral errors. This made the actuator configurations difficult to achieve, and the path-following controller was unable to achieve a steady state condition. The implementation of anti-saturation techniques was not sufficient to mitigate these problems. On the other hand, because the GPS positioning has errors itself, the controller is set to start tracking the next waypoint when the USV arrives within the specified range of the tracking waypoint. Therefore, when the error between the actual heading and the desired heading is within a specific small range, the requirement of path-following is considered to be achieved. This makes the compensation for the steady state error unimportant in the path-following control of the USV. Hence, a PD controller, which is found to perform adequately in simulation and experiment, is used instead.

**Figure 13:**
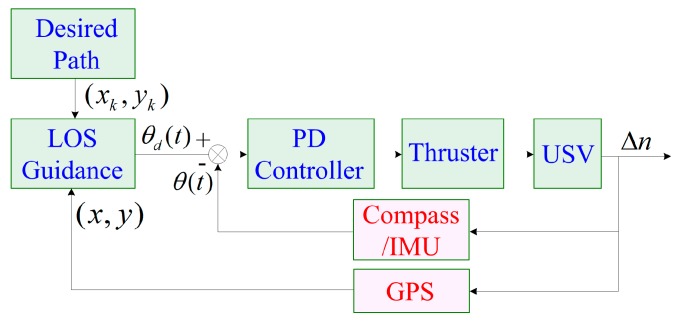
The USV motion control system.

The USV motion control system based on PD+LOS guidance is shown in Figure 13. According to the $\psi_{d}(t)$ obtained by the LOS algorithm, the heading error is defined as:

(38) $$e(t)=\psi (t)-\psi_{d}(t)$$

The output of the PD controller is the differential speed of the port and starboard thrusters, given by:

(39) $$\Delta n=K_{P}e(t)+K_{D}(e(t)-e(t-1))$$

In the PD controller, the import of the derivative part improves the dynamic characteristics of the system, but is particularly sensitive to noise. Therefore, the first order low-pass filter is added to the derivative part. The transfer function of the derivative filter can be expressed as $h(s)=\frac{1}{1+T_{f}s}$, $T_{f}$ is the filter time constant. Since the frequency of the input signal in the PD controller is 10 Hz, to reduce noise effects, $T_{f}$ is set to 0.1. The output of the PD controller after adding the derivative filter is given by

(40) $$\Delta n=K_{P}e(t)+\frac{T_{f}}{T_{f}+T}u_{D}(t-1)+\frac{K_{D}}{T_{f}+T}(e(t)-e(t-1))$$

where $u_{D}(t-1)$ is the output of the derivative part at the last moment. T is the period of the PD controller, which equals to 0.1 s.

Based on the established USV model and the PD+LOS control algorithm, we adjusted the PD parameters in simulation for a preliminary selection of PD parameters. The gains $K_{P}$ and $K_{D}$ of the PD controller were manually adjusted. The detailed steps were as follows: (1) we first set $K_{D}=0$, and adjusted $K_{P}$ gradually from small to large until the closed-loop response curve appeared equal amplitude oscillation; (2) we recorded the current value $K_{P}$ and the oscillation period $T_{o}$ of the closed-loop response curve. Based on the Ziegler-Nichols rules [39], we calculated the gains $K_{P}$ and $K_{D}$; (3) we then set PD parameters as the values calculated in step (2). Based on the system response, we optimized the PD parameters further. The initial selection of PD parameters in the simulation provided guidance for the setting of PD parameters in experiments, and reduced the corresponding adjustment workload. The finalized PD parameters were $K_{P}=5.3,\;K_{D}=5.6$. We conducted the simulations and experiments about path-following of the USV, including line path, circle path and zigzag path. Different from the line, circle and zigzag tests in system identification, the line path, circle path and zigzag path were closed loop maneuvers which were three tracking paths as examples generated by setting the desired waypoints. There were other paths such as rectangular path, eight-shaped path, etc. The three paths were served as examples to show the performance of the path-following. In addition, complicated time-dependent disturbances $\tau_{E}$ were considered as [33,40]:

(41) $$\{\begin{array}{c} \tau_{uE}=1+0.1\sin(0.1t)+0.3\cos(0.3t)\\ \tau_{vE}=1+0.2\sin(0.5t)+0.1\cos(0.2t)\\ \tau_{rE}=1+0.3\sin(0.2t)+0.1\cos(0.4t) \end{array}$$

**Figure 14:**
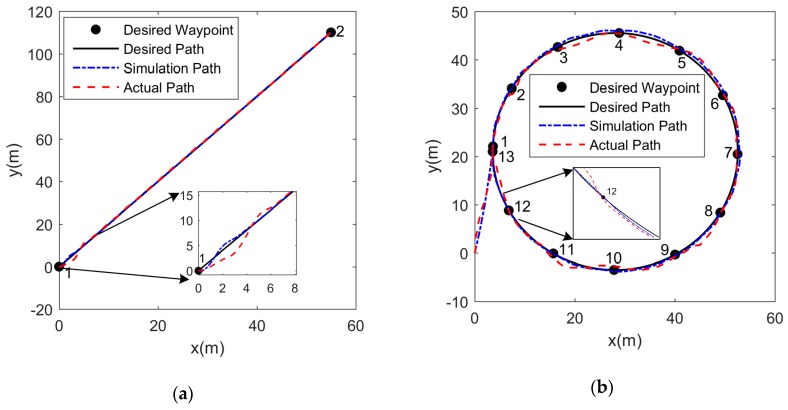

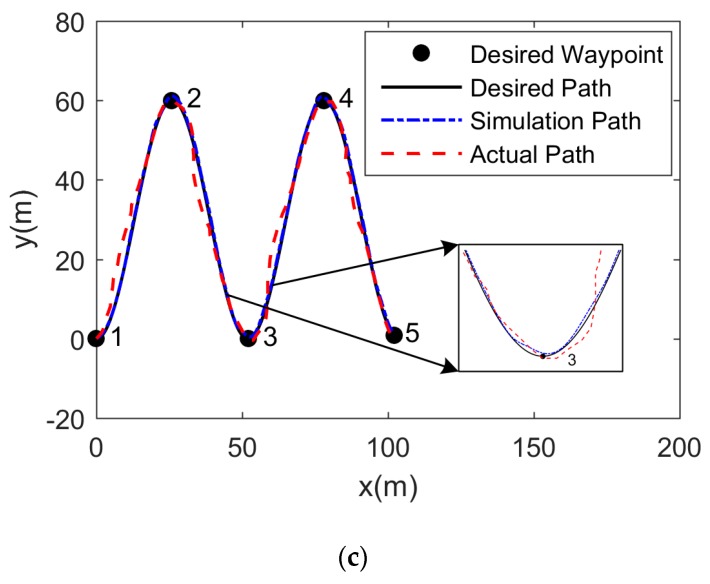
The simulation and experimental results of the simulation path, actual path and desired path: **(a)** line path; **(b)** circle path; **(c)** zigzag path.

**Figure 15:**
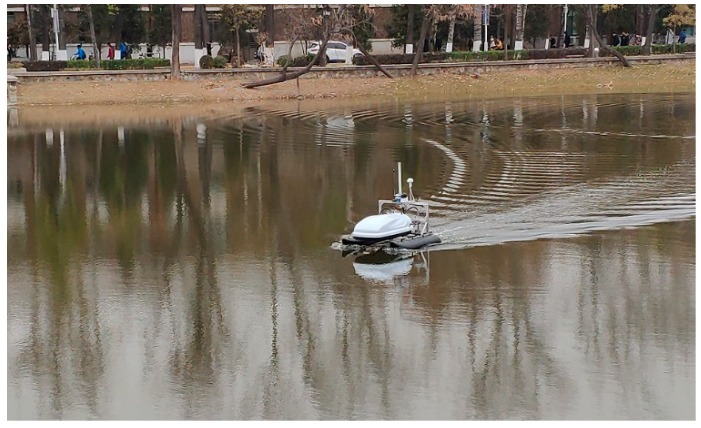
The experimental environment.

The time-dependent disturbances $\tau_{E}$ were added to the simulations of path-following. The conditions of simulations and experiments were set in the same. In the line path-following, the direction and the distance of the desired line path were identical. The starting direction and the radius of the desired circle path were the same in the circle path-following. In the zigzag path-following, the angle of each rotation and the duration of the desired zigzag path were identical. Besides, the initial headings of the USV in the three path-following were the same. Figure 14 gives simulation and experimental results for path following. The actual experimental environment of the lake is shown in Figure 15. The root mean square error (RMSE) of the distances between the desired waypoints and the actual positions of the USV is adopted to evaluate the performance of the controller, given by

(42) $$d_{RMSE}=\sqrt{\frac{\sum_{i=1}^{n}\left({\left(x_{i}-x_{di}\right)}^{2}+{\left(y_{i}-y_{di}\right)}^{2}\right)}{n}}$$

where $\left(x_{di},y_{di}\right)$ is the position of the *i* desired waypoint. *n* is the number of the desired points. $\left(x_{i},y_{i}\right)$ is the *i* actual position of the USV.

As shown in Figure 14a, the distance error is large when starting to track the desired waypoint. However, the deviation is corrected by the adjustment of the controller, and the path-following performance is high in the latter half of the line path. In the circle and zigzag path-following, the RMSEs of the distances between the desired waypoints and the actual positions are larger than the RMSEs in the line path-following, both in simulations and experiments. It indicates that the performance of the controller in a straight line path-following is better than that in curve path-following. However, because the RMSEs in the three path-following are all small, the controller still maintains excellent performance in both line and curve path-following. Due to the existence of disturbances in simulations and experiments in Figure 14, there are distance deviations between the simulation path, actual path and the desired path. Moreover, the RMSEs in the experiments of line, circle, and zigzag path-following are 0.33 m, 0.86 m, 0.54 m, respectively. They are larger than those in the simulations, which are 0.25 m in line path, 0.57 m in circle path, 0.42 m in zigzag path. Considering the accuracy of 1 m of GPS positioning, the distance deviations in simulations and experiments can be accepted in practice. Therefore, clearly satisfactory path following has been achieved by the USV in both simulations and experiments.

## 6. Conclusions

The motion control problem for USVs has been studied with a focus on those equipped with rudderless double thrusters due to their simple mechanical structure, flexible steering capability and some other associated benefits. In particular, a propeller thrust model was derived first, with only two unknown parameters, which reduces the workload of system identification. Secondly, a three-DOF dynamic model was established, which combines the propeller thrust model to represent the thrust in more detail. Moreover, the propulsion of the USV is provided by the resultant force generated by the port and starboard thrusters completely, and the rotational moment is related to the differential thrust and the distance of two thrusters. It provides guidance for the modeling of general USVs with rudderless double thrusters. Then, the model parameters were identified and verified through some standard motion measurements, including the acceleration test, circle test and zigzag test. Finally, based on the dynamic model, the PD+LOS control algorithm was employed to perform the path-following control of the USV. The parameters in the PD controller were initially selected based on simulations and then tuned during experimental tests, which reduced the corresponding adjustment workload in the experiments. The main goal of this work is to establish and identify a three-DOF dynamic model for the USV with rudderless double thrusters and validate the performance of the USV concerning the established model through simulations and experiments. Through a series of simulations and experiments, the RMSEs of distances between the desired waypoints and the actual positions in path-following were small, which demonstrated that the motion control of the USV was achieved successfully. Moreover, the controller could maintain good performance in both line and curve path-following. It indicated the validity and practical value of the established models. It links theory with practice, and the established model provides guidance for the experimental tests.

## Figures and Tables

**Table 1:** Physical characteristics of the FAS-01 USV.

Parameter	Value
Length overall (LO)	1.5 m
Waterline length (L)	1.3 m
Draft (D)	0.125 m
Beam overall (W)	0.9 m
Diameter of the propeller	0.27 m
Distance between two propellers (B)	0.52 m
Mass	50 kg
USV velocity (max)	1.5 m/s

**Table 2:** The corresponding thrust values under different motor commands.

Motor Command (%)	−100	−90	−80	−70	−60	−50	−40	−30	30	40	50	60	70	80	90	100
Thrust (N)	**−110**	**−88**	**−64**	**−45**	**−30**	**−14**	**−8**	**−1**	2	9	15	39	54	120	177	227

**Table 3:** Five combinations of motor commands in the zigzag test.

Test	High Value (%)	Low Value (%)
1	100	80
2	90	75
3	85	65
4	75	57
5	65	50
